# The GlyT1 Inhibitor Bitopertin Ameliorates Allodynia and Hyperalgesia in Animal Models of Neuropathic and Inflammatory Pain

**DOI:** 10.3389/fnmol.2017.00438

**Published:** 2018-01-10

**Authors:** Anja Armbruster, Elena Neumann, Valentin Kötter, Henning Hermanns, Robert Werdehausen, Volker Eulenburg

**Affiliations:** ^1^Institute of Biochemistry, Emil-Fischer-Center, University of Erlangen-Nuremberg, Erlangen, Germany; ^2^Department of Anesthesiology, Medical Faculty, Heinrich-Heine-University, Düsseldorf, Germany; ^3^Department of Anesthesiology, Academic Medical Center, Amsterdam, Netherlands

**Keywords:** neuropathic pain, bitopertin, glycine transporter, glycinergic inhibition

## Abstract

**Background:** Chronic pain conditions are difficult to treat and the therapeutic outcome is frequently unsatisfactory. Changes in excitation/inhibition balance within the dorsal horn contribute to the establishment and persistence of chronic pain. Thus, facilitation of inhibitory neurotransmission is a promising approach to treat chronic pain pharmacologically. Glycine transporter 1 (GlyT1) plays an important role in regulating extracellular glycine concentrations. Aim of the present study therefore was to investigate whether the specific GlyT1 inhibitor bitopertin (RG1678; RO4917838) might constitute a novel treatment for chronic pain by facilitating glycinergic inhibition.

**Methods:** Mechanical allodynia and thermal hyperalgesia were induced by chronic constriction injury of the sciatic nerve or carrageenan injections into the plantar surface of the hind paw in rodents. The effect of acute and long-term bitopertin application on the reaction threshold to mechanical and thermal stimuli was determined. General activity was determined in open field experiments. The glycine concentration in cerebrospinal fluid and blood was measured by HPLC.

**Results:** Systemic application of bitopertin in chronic pain conditions lead to a significant increase of the reaction thresholds to mechanical and thermal stimuli in a time and dose-dependent manner. Long-term application of bitopertin effectuated stable beneficial effects over 4 weeks. Bitopertin did not alter reaction thresholds to stimuli in control animals and had no effect on general locomotor activity and anxiety but lead to an increased glycine concentration in cerebrospinal fluid.

**Conclusion:** These findings suggest that inhibition of the GlyT1 by bitopertin represents a promising new approach for the treatment of chronic pain.

## Introduction

Chronic pain is defined as a long-lasting pain which outlasts the period of healing of the original noxious stimulus (Kuner and Flor, 2016). Maladaptive processes within the somatosensory system cause activation patterns within the CNS that are different from those observed after acute painful stimuli (Moxon et al., 2014; Kuner and Flor, 2016). It has been suggested that chronic pain depending on its source involves different adaptive processes within the somatosensory system. Here, chronic pain associated by inflammation was shown to depend on a protein kinase A (PKA)-dependent downregulation of predominantly glycine receptor α3 mediated glycinergic neurotransmission (Harvey et al., 2004), whereas neuropathic pain is independent of these processes (Hosl et al., 2006). Consistent with these differences the therapeutic strategies for the treatment of the assorted chronic pain modalities varie. Especially for neuropathic pain, treatment options are insufficient and therapeutic outcome is deficient in many cases (Baron et al., 2010). Treatment guidelines for chronic neuropathic pain include the treatment with tricyclic antidepressants, dual norepinephrine/serotonin reuptake inhibitors Ca^2+^ α_2_δ inhibitors and opioids (Gilron et al., 2015). Adverse effects, caused by the central nervous action of most of these substances, limit their clinical use and commonly prevent a sufficient treatment (Kremer et al., 2016). Thus, the development of new therapeutic strategies for the treatment of chronic pain is urgently required. It is widely accepted that diminished inhibitory neurotransmission within the dorsal horn of the spinal cord is a major contributor to the establishment of many chronic pain conditions (Zeilhofer, 2008). Based on this assumption the pharmacological facilitation of GABAergic or glycinergic inhibitory neurotransmission in the dorsal horn is considered a promising strategy for the treatment of chronic pain. The use of subtype specific GABA_A_ receptor mimetics has been shown to be difficult due to the high abundance of GABA_A_ receptors in higher brain regions. Despite some modulatory effects in higher brain regions, glycinergic inhibition is largely restricted to the caudal nervous system and is therefore considered an attractive therapeutic target for pharmacological intervention.

Here, the inhibition of neurotransmitter transporter proteins offers the advantage of an activity-dependent modulation of individual synapse efficacy (Eulenburg and Gomeza, 2010). Consistent with this idea it was shown that inhibition of GlyT1 can facilitate both glycine dependent inhibitory neurotransmission via Glycine receptors (GlyRs) and glutamatergic neurotransmission via *N*-Methyl-D-aspartate receptors (NMADRs). We have shown previously that the lidocaine metabolite *n*-ethylglycine (EG), a GlyT1 substrate, has antihyperalgesic and antiallodynic properties, most likely via an elevation of glycine concentration within the extracellular space (Werdehausen et al., 2012; Werdehausen et al., 2015). Due to the relative low affinity to GlyT1 and unfavorable pharmacokinetic properties this substance is most likely not a suitable agent for routine application in pain therapy. Here, recently developed novel GlyT1 inhibitors like bitopertin (Hoffmann–La Roche) or BI 425809 (Boehringer–Ingelheim) might provide novel tools to facilitate glycine dependent neurotransmission in the context of chronic pain.

In this study, we analyzed the effect of the GlyT1 inhibitor bitopertin on pathologically increased pain responses. The highly potent and selective GlyT1 inhibitor bitopertin has been initially developed for the treatment of schizophrenia (Javitt, 2012). Here, increasing the extracellular glycine concentrations GlyT1 inhibitors was predicted to counteract schizophrenia by facilitating the efficacy of *N*-methyl-D-aspartate receptors (NMDAR). In phase II clinical trials, bitopertin showed promising results at intermediate doses (Umbricht et al., 2014), suggesting a bell-shaped dose-response curve of the bitopertin effect. Phase III trials, however, failed to reveal an additional benefit of bitopertin when compared to placebo (Bugarski-Kirola et al., 2014, 2016a; Hirayasu et al., 2016). In all studies bitopertin showed a high bioavailability after oral application, and severe adverse effects were not reported. Here we show that bitopertin ameliorates the facilitated pain response in animal models of neuropathic and inflammatory pain, whereas acute pain was not affected. Consistent with previous clinical trials using bitopertin, no severe side effects were observed. Taken together, our data suggest that bitopertin constitutes a new option for the treatment of chronic pain conditions.

## Materials and Methods

### Reagents

Bitopertin (RG1678) was provided by Hycultec GmbH (Beutelsbach, Germany). All other reagents were purchased from Sigma–Aldrich (St. Louis, MO, United States), Carl Roth (Karlsruhe, Germany) or Applichem (Darmstadt, Germany). For the intraperitoneal (i.p.) application in mice, bitopertin was dissolved in 2-hydroxypropyl-β-cyclodextrin 30%, for the long-term application via osmotic minipumps in polyethylene glycol 400. Gabapentin was dissolved in saline. For the oral (p.o.) and subcutaneous (s.c.) application experiments in rats bitopertin was dissolved in dimethylsulfoxide (50 mg ml^-1^) and diluted in polysorbate 80 solution (0.3%, pH 6.4 NaOH).

### Animals

All experiments were performed in accordance with animal welfare regulations and approved by local authorities. Adult male Wistar rats (weight 247 ± 30 g) and adult male C57BL/6J mice (weight 27 ± 3 g) were housed in controlled environment [22 ± 2°C, humidity 50 ± 5%, a 12 h/12 h light/dark schedule, standard food pellets (Ssniff Spezialdiäten GmbH, Germany) and water available *ad libitum*]. After the last measurement, the animals were killed by i.p. injection of pentobarbital (Merial, Hallbergmoos, Germany) (rats) or by CO_2_ (mice).

### Animal Models for Neuropathic and Inflammatory Pain

For the induction of neuropathic pain in rats, chronic constriction injury (CCI) was induced under general pentobarbital anesthesia (60 mg kg^-1^, i.p.) using four loose ligatures (3-0 catgut). Due to the relative stiffness of the catgut ligatures that made a proper application of the ligature difficult on the relatively small sciatic nerve from mice, silk ligature was used for the induction of neuropathic pain in mice. Here, the left sciatic nerve was loosely ligated by three ligatures (8-0 Sofsilk^®^) under general anesthesia using isoflurane (Bennett and Xie, 1988). After application of the ligatures a slowly developing hyperalgesia and allodynia was observed, consistent with the establishment of neuropathic pain. Inflammatory pain was induced by s.c. injection of carrageenan 1% 25 μl (kappa/lambda) into the plantar surface of the left hind paw under general isoflurane anesthesia.

For bolus applications, the GlyT1 inhibitor bitopertin or vehicle was applied p.o. by a gavage or by i.p. or s.c. injections. For long-term treatment, bitopertin or vehicle was applied via Alzet^®^ osmotic minipumps, model 1004 (Charles River Laboratories, Sulzfeld, Germany), implanted s.c. at the lower neck of the animals. The applicator was blinded to the randomized treatment group assignment.

### Behavioral Tests

Sensory testing was performed before CCI or carrageenan injection, and after development of the pain phenotype (10 days after CCI for mice, 11–15 days after CCI for rats and 3 h after carrageenan injection). Mechanical and thermal reaction thresholds were determined with a plantar aesthesiometer and a modified Hargreaves apparatus (Ugo Basile, Comerio, Italy), respectively, at the indicated time points on the affected hind paw. The contralateral side was used as control. Paw thickness before and after carrageenan treatment was measured using a thickness gauge (Model No. 7313; Mitutoyo Corporation, Kawasaki, Japan). For open field experiments, mice were put in an open field arena (50 cm × 50 cm × 50 cm, gray plastic) for 60 or 120 min and their activity was determined by a video tracking system (VideoMot; TSE Systems, Bad Homberg, Germany). All experiments were performed blinded to the randomized treatment group assignment.

### High-Pressure Liquid Chromatography

Cerebrospinal fluid samples were drawn from the cisterna magna in adult male C57BL/6J mice as described (Liu and Duff, 2008). Blood samples were collected from the facial vein using heparinized capillaries. The glycine concentrations in cerebrospinal fluid and blood were determined using high pressure liquid chromatography (HPLC) as described (Kurolap et al., 2016).

### Hemoglobin Determination

Blood samples were collected from the facial vein using heparinized capillaries. The sample was mixed with 2 ml cyanide-solution (K_3_[Fe(CN)_6_] 0.6 mM, KCN 0.75 mM) and the hemoglobin concentration was measured photometrically at a wavelength of 546 nm.

## Results

### Single-Dose Bitopertin Ameliorates Neuropathic Pain in Mice without Affecting Motor Activity or Acute Pain

**FIGURE 1:**
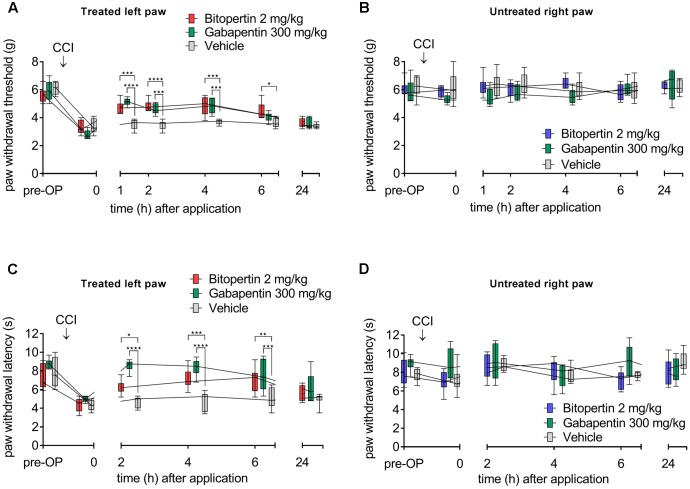
A single dose of bitopertin reduces mechanical allodynia **(A)** and thermal hyperalgesia **(C)** after induction of neuropathic pain by chronic constriction of the sciatic nerve (CCI) in mice. Bitopertin (2 mg kg^-1^ i.p.), gabapentin (300 mg kg^-1^ i.p.) or vehicle was applied 10 days after CCI and mechanical allodynia (Plantar Aesthesiometer) and thermal hyperalgesia (Hargreaves apparatus) were determined at the indicated time points by means of the paw withdrawal thresholds. At the untreated right paw, no effect after application of bitopertin or gabapentin was observed **(B,D)**. Data are expressed as box and whiskers ± min to max, *n* = 6 per group (two-way ANOVA and Bonferroni test, ^∗^*P* < 0.05, ^∗∗^*P* < 0.01, ^∗∗∗^*P* < 0.001, ^∗∗∗∗^*P* < 0.0001).

To test if bitopertin ameliorates the facilitated pain response in animal models for chronic pain, neuropathic pain was induced in mice by CCI. Sensory testing revealed the development of allodynia and hyperalgesia within 10 days after CCI on the ipsilateral side. No changes were observed on the contralateral control side (**Figure 1**) or after sham operations (data not shown). Initially, a bolus application of 2 mg kg^-1^ (i.p.) was tested, which was shown in previous pharmacokinetic studies not to cause any severe side effects, like motor impairment or respiratory distress (Pinard et al., 2010). For comparison, gabapentin (300 mg kg^-1^ i.p.) was used, which was previously shown to efficiently ameliorate hyperalgesia and allodynia (Field et al., 1997; Tanabe et al., 2005). Here, both the application of bitopertin or gabapentin but not of vehicle alone, caused a significant increase in the reaction threshold to mechanical stimuli (**Figure 1A**) 1–6 h after substance application. 24 h after bitopertin or gabapentin application, the reaction threshold was indistinguishable from that of animals, which received a vehicle injection only.

Bitopertin also ameliorated thermal hyperalgesia induced by CCI (**Figure 1C**), although gabapentin showed an apparent faster and stronger reduction of the hyperalgesia induced by CCI. Here, bitopertin induced a significant increase in the reaction threshold within 2–6 h with the maximal effect observed 6 h after application. In contrast, gabapentin showed its strongest effect already after 2 h which slowly declined after a plateau of 2 h. However, 24 h after application of either substances, the reaction threshold was indistinguishable from that of animals that received vehicle only (**Figure 1C**). No difference in the reaction threshold of the contralateral side between bitopertin and vehicle treated animals was observed, regardless of the stimulation paradigm (**Figures 1B,D**). A reduction of the bitopertin concentration to 0.2 mg kg^-1^ still resulted in a significant increase of both the reaction threshold to mechanical stimuli (4.8 ± 0.4 g vs. 3.7 ± 0.1 g for bitopertin vs. vehicle treated animals, *p* < 0.02, two way ANOVA with Bonferroni *post hoc* comparison, *n* = 4), and amelioration of the CCI induced thermal allodynia (9.7 ± 2.7 s vs. 4.9 ± 0.95 s for bitopertin vs. vehicle treated animals, *p* > 0.01, two way ANOVA with Bonferroni *post hoc* comparison, *n* = 4), 2 h after application of the substance.

### Single-Dose Bitopertin Also Reduces Allodynia and Hyperalgesia in Inflammatory Pain in Mice

**FIGURE 2:**
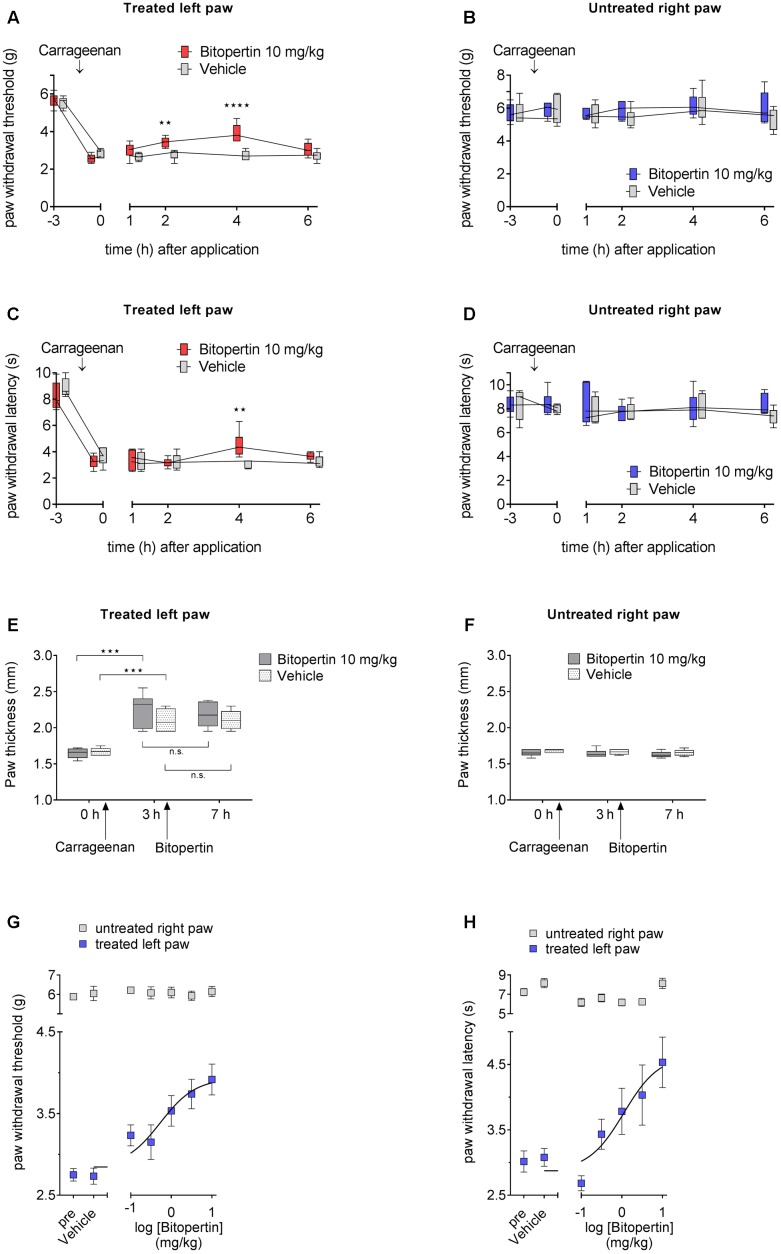
A single dose of bitopertin reduces mechanical allodynia **(A)** and thermal hyperalgesia **(C)** in inflammatory pain. Bitopertin (10 mg kg^-1^ i.p.) or vehicle was applied 3 h after induction of inflammatory pain by injection of 25 μl carrageenan (1%; kappa/lambda). Mechanical allodynia **(A)** and thermal hyperalgesia **(C)** was determined at the indicated time points with a plantar aesthesiometer and Hargreaves apparatus. At the untreated right paw no effect after application of bitopertin was observed **(B,D)**. **(E,F)** Inflammatory oedema was assessed by measuring the paw thickness of the treated **(E)** and untreated **(F)** control paw. Data are expressed as box and whiskers ± min to max, *n* = 6 per group (two-way ANOVA and Bonferroni test, ^∗∗^*P* < 0.01, ^∗∗∗^*P* < 0.001, ^∗∗∗∗^*P* < 0.0001). Dose response for the effect of bitopertin on paw withdrawal thresholds **(G)** and paw withdrawal latency **(H)** after induction of inflammatory pain and 4 h after application of bitopertin. Data are expressed as mean ± SEM, *n* = 6 per group.

To test if other forms of chronic pain are also susceptible for the treatment with bitopertin, we tested its effect in the carrageenan model of inflammatory pain. Within 3 h after injection of carrageenan, a pronounced oedema, as indicated by a significant increase in paw thickness was observed (**Figure 2E**). No oedemas were detectable in vehicle injected paws or on the contralateral side. The swelling of the paw was accompanied by a significant reduction in both thermal and mechanical stimuli-induced paw withdrawal threshold (**Figures 2A,C**), which is consistent with the establishment of a local inflammation. Subsequently, animals were treated with bitopertin (10 mg kg^-1^) or vehicle and repeatedly tested. In contrast to vehicle or untreated animals that did not show any significant changes in their reaction thresholds to mechanical stimulation over the observation period of 6 h, the reaction thresholds of bitopertin treated animals increased significantly after 2 h. This effect lasted until 4 h after injection of bitopertin. Six hours after bitopertin injection, the reaction thresholds between bitopertin and vehicle treated animals were indistinguishable (**Figure 2A**). After thermal stimulation, the differences between vehicle and bitopertin treated animals were less pronounced. Here, significant differences were only observed 4 h after bitopertin injection (**Figure 2C**). No differences in the reaction thresholds were observed at the contralateral side (**Figures 2B,D**), re-confirming that bitopertin does not affect sensory processing and reflex circuitry in naïve animals.

No effect on paw thickness was observed after bitopertin treatment (**Figures 2E,F**), supporting the hypothesis that the antihyperalgesic and antiallodynic effect of bitopertin is caused by a neuronal mechanism and is not a result of a possible antiphlogistic action.

We subsequently analyzed the dose-dependency of the antihyperalgesic and antiallodynic effect of bitopertin on inflammatory pain. Mice were treated with the indicated dose of bitopertin (0.1, 0.33, 1, 3.3, and 10 mg kg^-1^) and the reaction thresholds were determined 4 h after application. A clear dose-dependency of the antiallodynic and antihyperalgesic effect of bitopertin (**Figures 2G,H**) was observed, revealing an EC_50_ of 0.6 mg kg^-1^ for the antiallodynic effect and 1.1 mg kg^-1^ for the observed antihyperalgesic effect. Reaction thresholds at the contralateral side to mechanical or thermal stimuli did not reveal any significant changes regardless of the bitopertin concentrations used.

**FIGURE 3:**
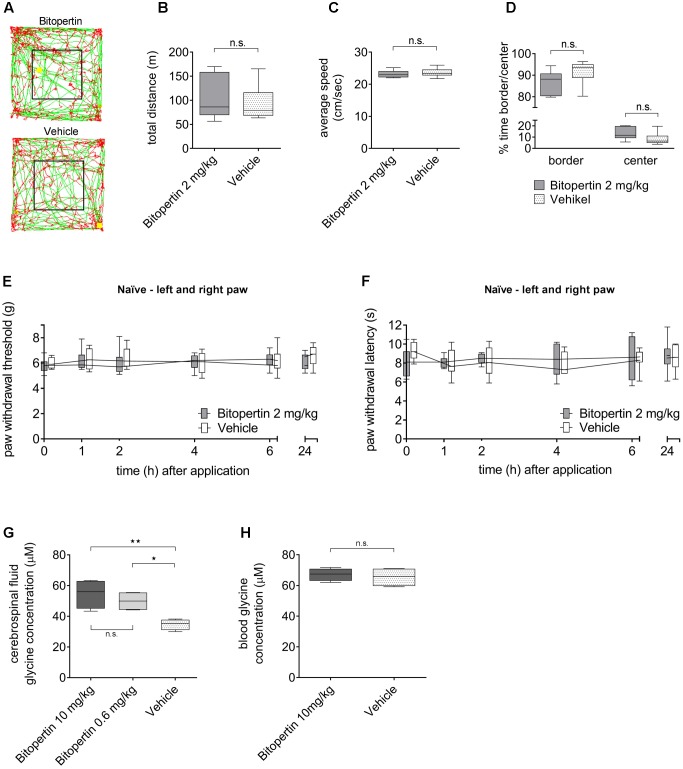
A single dose of bitopertin has no effect on motor activity or sensory processing and leads to an increased glycine concentration in cerebrospinal fluid without affecting the glycine concentration in the blood. Behavioral data from naïve adult mice after receiving a single dose of bitopertin (2 mg kg^-1^ i.p.) or vehicle. Open field experiments were performed 2 h after application of bitopertin for 120 min. **(A)** Original traces from the first 10 min. Total distance **(B)**, average speed **(C)** and time spent in the periphery/center **(D)** were determined. Data are expressed as box and whiskers ± min to max, *n* = 6 per group; no significant differences vs. control group (vehicle) were detected (Student *t*-test, *P* ≥ 0.05). Effect of bitopertin on acute pain: Paw withdrawal thresholds to mechanical **(E)** and thermal **(F)** stimuli were determined with a plantar aesthesiometer or a modified Hargreaves apparatus at the indicated time points. Data are expressed as box and whiskers ± min to max, *n* = 4–5 per group; no significant differences vs. control group (vehicle) were detected (two-way ANOVA and Bonferroni test, *P* ≥ 0.05). Cerebrospinal fluid **(G)** and blood **(H)** samples were drawn 2 h after application of bitopertin (10 mg kg^-1^ i.p.) and the glycine concentration was determined using high-pressure liquid chromatography. Data are expressed as box and whiskers ± min to max, *n* = 4-5 for each group (duplicate for each sample) (one-way ANOVA and Student *t*-test, ^∗^*P* < 0.05, ^∗∗^*P* < 0.01).

To determine possible effects of systemic bitopertin treatment on the general behavior, we treated naïve adult mice with bitopertin (2 mg kg^-1^ i.p.) or vehicle, respectively, and analyzed their behavior in an open field experiment 2 h after injection (**Figure 3**). Here, bitopertin had no influence on the total distance and the average speed of the animals (**Figures 3B,C**). Also, the ratio of the time spent in the center or border region was not different (**Figure 3D**).

To test if bitopertin affects general sensory information processing, the reaction threshold to mechanical and thermal stimuli was also analyzed in naïve animals after bitopertin treatment (**Figures 3E,F**). No differences between animals treated with bitopertin (2 mg kg^-1^ i.p.) and vehicle treated animals were observed, regardless of the mode of stimulation.

### Bitopertin Increases the Glycine Concentrations in Cerebrospinal Fluid But Not in Blood

The effect of bitopertin treatment on the blood and CSF glycine concentration was determined in naïve mice using a HPLC based analysis. Vehicle or bitopertin (10 or 0.6 mg kg^-1^, i.e., the highest concentration tested and the resulting EC_50_ determined in the inflammatory pain model) were injected (i.p.) at the indicated concentrations into naïve mice and blood and CSF samples were collected 2 h after injection. Here, CSF samples obtained from bitopertin treated animals showed a significantly elevated glycine concentration as compared to vehicle treated animals, whereas the blood glycine concentration was indistinguishable between all groups (**Figures 3G,H**).

### Long-term Application of Bitopertin Maintains the Beneficial Effects on Neuropathic Pain in Mice without Affecting Motor Activity, Acute Pain, or Hemoglobin Level

**FIGURE 4:**
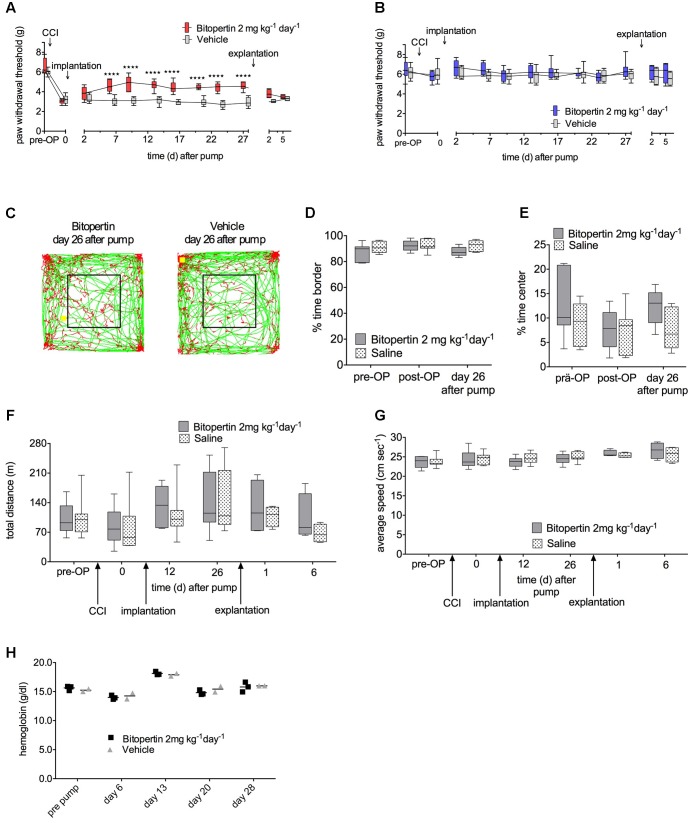
Long-term application of bitopertin reduces mechanical allodynia and thermal hyperalgesia after induction of neuropathic pain by CCI in mice over 4 weeks and has no effect on the hemoglobin level. Bitopertin (2 mg kg^-1^ d^-1^) or vehicle was applied using Alzet^®^ osmotic minipumps starting 10 days after CCI. Mechanical reaction thresholds of the treated left paw were determined with a plantar aesthesiometer at the indicated time points **(A)** and untreated right paw **(B)**. Data are expressed as box and whiskers ± min to max, *n* = 8 per group; (two-way ANOVA and Bonferroni test, ^∗∗∗∗^*P* < 0.0001). Open field experiments were performed naïve, after CCI, after implantation of the pump and after explantation of the pump each time for 60 min. **(C)** Original traces from the first 10 min of the open field 26 days after implantation. Time spent in the periphery **(D)**, time spent in the central quadrant **(E)**, total distance **(F)**, and average speed **(G)** were determined. Data are expressed as box and whiskers ± min to max, *n* = 8 per group; no significant differences vs. control group (vehicle) were detected (two-way ANOVA and Bonferroni test, *P* ≥ 0.05). **(H)** Hemoglobin level was determined before and 6, 13, 20, and 28 days after implantation of the pump. Data are expressed as scatter dot plot, the mean is indicated, *n* = 3 for the bitopertin group and *n* = 2 for the vehicle group; no significant differences vs. control group (vehicle) were detected (two-way ANOVA and Bonferroni test, *P* ≥ 0.5).

First, we tested whether a prolonged continuous application of bitopertin results in a long-lasting amelioration of neuropathic pain in mice. After unilateral CCI-surgery and establishment of hyperalgesia and allodynia, osmotic minipumps which provided a constant supply of bitopertin or vehicle, respectively, over a time course of 4 weeks (2 mg kg^-1^ d^-1^) were implanted. Sensory testing and open field experiment were performed every 3–7 days. Directly after implantation, there was no significant difference between animals that received pumps filled with bitopertin or vehicle. Five days after implantation, a significant increase in the reaction threshold to mechanical stimulation was observed, that remained as long as the pumps remained implanted. After explantation, reaction threshold differences between treatment groups were decreased after 2 days. Five days after explantation, the reaction thresholds of both groups were indistinguishable (**Figure 4A**). There was no effect on the reaction thresholds on the contralateral side during the whole testing period (**Figure 4B**). In open field experiments, neither the total distance traveled nor the speed of movement or ratio of time spend in the periphery and center, was different between animals receiving bitopertin or vehicle (**Figures 4C–G**). These data demonstrate that upon prolonged application, bitopertin can exert a long-lasting amelioration of allodynia in rodent models for neuropathic pain. Furthermore, the hemoglobin level was determined before and once per week after implantation of the pump. Here, no significant differences were observed between the bitopertin and vehicle group (**Figure 4H**).

**FIGURE 5:**
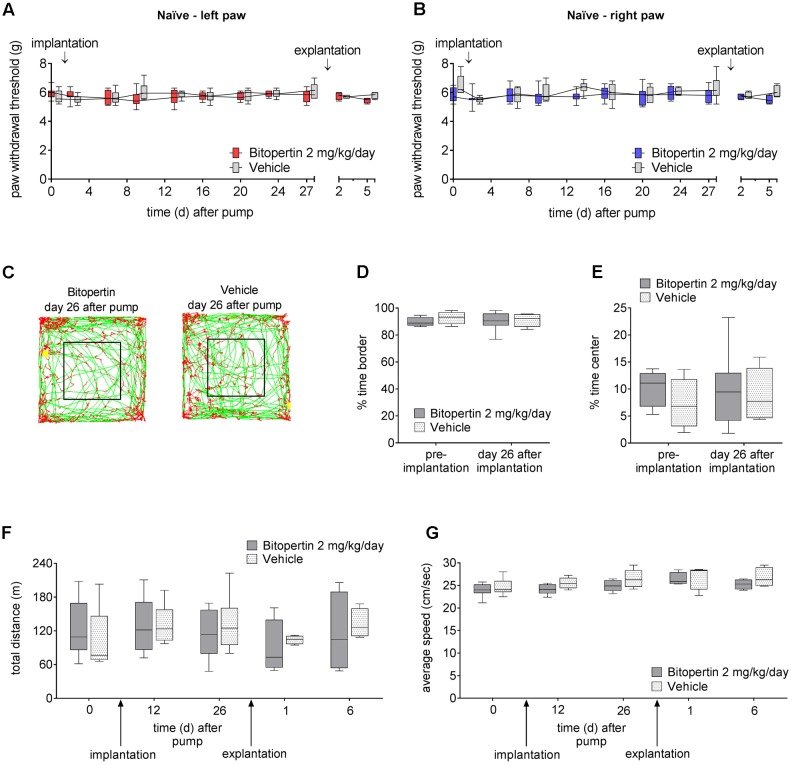
Long-term application of bitopertin has no effect on motor activity or acute pain response. Behavioral data from naïve adult mice during and after long-term application of bitopertin (2 mg kg^-1^ d^-1^) or vehicle by Alzet^®^ osmotic minipumps. Mechanical reaction thresholds of the left paw **(A)** and right paw **(B)** were determined with a plantar aesthesiometer at the indicated time points. Data are expressed as box and whiskers ± min to max, *n* = 6–8 per group; no significant differences vs. control group (vehicle) were detected (two-way ANOVA and Bonferroni test, *P* ≥ 0.05). Open field experiments were performed naïve, after implantation of the pump and after explantation of the pump each time for 60 min. **(C)** Original traces from the first 10 min of the open field 26 days after implantation. Time spent in the periphery **(D)**, time spent in the central quadrant **(E)**, total distance **(F)**, and average speed **(G)** were determined. Data are expressed as box and whiskers ± min to max, *n* = 6–8 per group; no significant differences vs. control group (vehicle) were detected (two-way ANOVA and Bonferroni test, *P* ≥ 0.05).

In order to identify subtle effects of prolonged bitopertin application, bitopertin or vehicle filled minipumps were implanted into naïve animals. Here, no significant differences in the reaction threshold to mechanical stimuli or in open field experiments were observed between animals receiving bitopertin or vehicle before, during or after the application period (**Figure 5**).

### Single-Dose Bitopertin Also Ameliorates Neuropathic Pain in Rats

**FIGURE 6:**
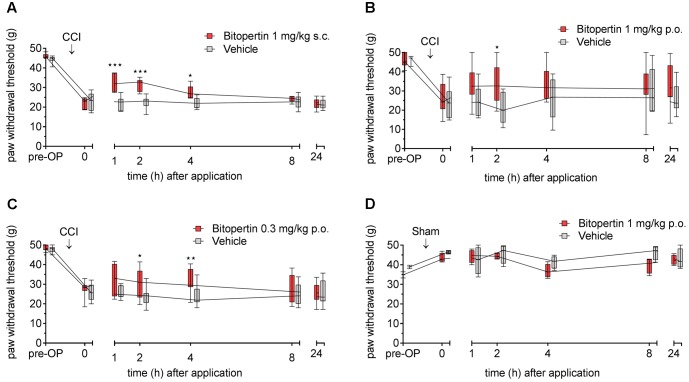
Bitopertin ameliorates allodynia in rats after parenteral **(A)** and oral application **(B,C)**. Allodynia was induced in adult male Wistar rats by CCI of the sciatic nerve. In sham-operated animals **(D)** the sciatic nerve was only exposed but no ligature was applied. 11–15 days after the operation reaction threshold to mechanical stimuli was determined. After application of bitopertin or vehicle, respectively, the reaction threshold to mechanical stimuli was determined at the indicated time points. Data are expressed as box and whiskers ± min to max, *n* = 6 per group **(A)**, *n* = 13 for bitopertin and *n* = 6 for vehicle **(B)**, *n* = 9 for bitopertin and *n* = 11 for vehicle **(C)**, *n* = 4 per group **(D)** (two-way ANOVA and Bonferroni test, ^∗^*P* < 0.05, ^∗∗^*P* < 0.01, ^∗∗∗^*P* < 0.001).

We next tested if the beneficial effect of bitopertin is also present in another rodent species. Therefore, allodynia was induced in rats by unilateral CCI. After 11–15 days, a significantly altered reaction threshold to mechanical stimuli was observed. Bitopertin (1 mg kg^-1^) or vehicle were injected s.c. and the reaction threshold to mechanical stimuli was determined at the indicated time points. In this setting, the application of 1 mg kg^-1^ bitopertin resulted in a significant but transient amelioration of the observed allodynia, whereas vehicle application had no effect. The antiallodynic effect was already observable 1 h after injection. After 2 h, the reaction threshold slowly declined, and after 24 h vehicle and bitopertin treated animals were again indistinguishable (**Figure 6A**). Additionally, we tested if the antiallodynic effect of bitopertin was also achievable after oral application. Here, a dose of 1 mg kg^-1^ had a similar effect on the reaction threshold to mechanical stimuli, (**Figure 6B**). A reduced dose of 0.3 mg kg^-1^ still lead to significantly increased withdrawal thresholds 2 and 4 h after application (**Figure 6C**), whereas the normal processing of mechanosensation acute pain as analyzed in sham-operated animals was not affected (**Figure 6D**).

## Discussion

Current medications of chronic pain conditions allow only for an insufficient amelioration of the symptoms and cause severe side effects in many cases (Kremer et al., 2016). It has been demonstrated that the activity of glycinergic neurons modulates the perception of itch and pain already within the dorsal horn (Foster et al., 2015). Furthermore, in animal models of inflammatory pain, inhibition of the glycine receptor (GlyR) subunit α3 mediated by prostaglandin E2 (PGE2) induced phosphorylation has been shown to effectuate pain hypersensitivity (Harvey et al., 2004). Consistently, a recently identified phosphorylation-specific modulator of GlyR α3 (Acuña et al., 2016) ameliorated inflammatory pain. Despite these recent progresses, the pharmacology of the GlyRs, however, is still insufficiently developed.

Thus, the indirect targeting of GlyRs by modulating the extracellular glycine concentrations is an attractive alternative. The extracellular concentration of glycine within the CNS is synergistically regulated by GlyT1 and GlyT2. Consistently, inhibitors for both transporters were shown to cause a significant increase in the CSF glycine concentration (Whitehead et al., 2004; Eulenburg et al., 2005). Moreover, inhibition of either transporters have been shown to have some antihyperalgesic and antiallodynic potential. Here, inhibition of GlyT2 by the inhibitors ALX1393 and ORG 25543 were effective at least at intermediate doses (Morita et al., 2008). Complete inhibition of GlyT2, however, was shown to results in a rundown of glycinergic neurotransmission most likely due to a breakdown in the presynaptic transmitter supply (Bradaia et al., 2004). Unfavorable pharmacokinetic properties of these GlyT2 inhibitors, however, prevented a clinical application. Future studies, analyzing the *in vivo* properties of newly developed GlyT2 inhibitors (Mingorance-Le Meur et al., 2013; Mostyn et al., 2017) might revive the interest in GlyT2 inhibitors for the treatment of chronic pain in the future.

Inhibition of the glycine uptake activity by GlyT1 inhibitors like ALX5407, ORG 25935, or artificial substrates like EG or sarcosine was shown to exert an antiallodynic and/or antihyperalgesic effect in animal models for chronic pain (Hermanns et al., 2008; Morita et al., 2008; Barthel et al., 2014). A clinical application of ALX5407 or ORG 25935, however, is precluded by their practically irreversible mode of GlyT1 inhibition that cause severe long lasting side effects like hypotonia and respiratory depression if used at high concentrations (Perry et al., 2008 and data not shown). Bitopertin has been extensively tested and its good safety profile is well documented by three large phase III clinical trials in the context of psychosis (Bugarski-Kirola et al., 2014, 2016a,b). Noteworthy, a mild but tolerable effect on the hemoglobin level was observed in animal studies as well as in humans, (Winter et al., 2016) possibly due to expression of GlyT1 in reticulocytes. In our study, no changes in the hemoglobin level during the bitopertin treatment (2 mg kg^-1^ d^-1^) were observed even after prolonged treatment periods over 4 weeks. This discrepancy might result from differences in the dosing regimen (bolus vs. continuous application) or species differences.

In our animal models for inflammatory and neuropathic pain bitopertin showed beneficial effects, whereas the perception of acute pain was not affected. Here, especially in the neuropathic pain model, already doses below 1 mg kg^-1^ were sufficient to elicit significant antiallodynic and antihyperalgesic effects both in rats (see **Figure 6**) and mice (**Figures 2G,H**). In contrast to gabapentin, that had similar effects on thermal hyperalgesia and mechanical allodynia, the effect of bitopertin on thermal hyperalgesia was only weak, albeit significant and showed a slower kinetic as compared to its effect on the CCI-induced mechanical allodynia. These findings contrast findings using the artificial substrate EG. Here, a single application ameliorated efficiently both thermal hyperalgesia and mechanical allodynia with approximately similar kinetics (Werdehausen et al., 2015). These differences might result from differences between artificial substrates that also allow for an induction of GlyT1 mediated glycine release from GlyT1 expressing cells, and GlyT1 specific antagonists. Evidence that GlyT1 substrates like sarcosine and inhibitors of GlyT1 indeed have diverging effects on CNS function despite comparable effects of the extracellular glycine concentration come from studies in the context of psychosis. Here, add-on treatment of patients with a high dose of sarcosine resulted in a significant amelioration of especially negative symptoms (Strzelecki et al., 2015), whereas bitopertin (at least in the Phase III clinical trials) was shown to be not effective (Bugarski-Kirola et al., 2016a). Whether these differences are a result of the different mechanism of action on GlyT1 or a result of the different substrate specificity – sarcosine, e.g., functions as a partial agonist on GlyRs, as well as NMDAR, but also possibly effects system A (Javitt et al., 2005) and system N (Hamdani et al., 2012) – is unclear at present, and requires further studies.

The antihyperalgesic effect observed after single application of bitopertin was observable for 4–6 h, which is in agreement with the pharmacokinetic properties of bitopertin in rodents (*t*_1/2_ = 4–6 h) (Pinard et al., 2010). In humans, the elimination of bitopertin, e.g., from plasma was estimated to be 52 h, suggesting that the beneficial effect on chronic pain after single application may possibly last longer in humans than in rodents.

Similar to previous experiments using GlyT1 inhibitors (Whitehead et al., 2004; Kurolap et al., 2016), treatment with bitopertin resulted a significant increase in the CSF glycine concentration was observed at doses as low as 0.6 mg kg^-1^, whereas the blood glycine concentration was not affected. Similar effects on the CSF glycine concentration were also described previously both in humans and rats (Alberati et al., 2012; Hofmann et al., 2016). Interestingly, no further increase in the extracellular glycine concentration was observed upon increase of the used bitopertin dose. These results are consistent with findings from mice with a more than 80% reduction in caudal GlyT1 expression and GlyT1 encephalopathy patients that also showed only moderately increased CSF glycine concentrations whereas blood glycine concentration were found unaltered (Kurolap et al., 2016). These findings suggest, that additional transporters, possibly with lower affinities preclude a further CSF specific increase of the extracellular glycine concentration. Whether these transporters include low affinity amino acid transporters like system A (Javitt et al., 2005) or system N (Hamdani et al., 2012) or antiporters like ASC-1 (Safory et al., 2015) is unclear at present and requires further studies. Although there was no apparent further increase in the CSF glycine concentration upon increasing the used bitopertin dose from 0.6 to 10 mg kg^-1^, there still was a significant increase in the antiallodynic and antihyperalgesic effect (compare **Figures 2G,H**, **3G**). These findings suggest that the local glycine concentrations in the dorsal horn of spinal cord, i.e., the brain region that are most likely responsible for the effect observed, differ from those determined in the CSF. Comparison of the effects observed by irreversible GlyT1 inhibitors like ALX5407 and bitopertin suggests that even at the highest bitopertin concentrations used, only a fraction of the total GlyT1 activity is blocked, whereas ALX5407 allows for an (almost) complete and long-lasting inhibition of GlyT1.

Whether the beneficial effect of bitopertin is a result of facilitated glycinergic, NMDAR-mediated glutamatergic neurotransmission, or both, however, remains unclear at present and requires further studies. Throughout our study we did not observe any adverse effects on motor activity as determined by open field analysis (see **Figures 3**, **4**) and RotaRod analysis (data not shown), general activity and/or anxiety as determined by openfield and elevated plus maze analysis, (see **Figures 3**, **4** and data not shown). These data are consistent with previous data describing that bitopertin is well tolerated at the investigated doses.

Our findings suggest that bitopertin might constitute an additional therapeutic option for the treatment of chronic pain conditions. Based on the good safety profile and the mild undesired effects observed so far, bitopertin might be suitable for both monotherapy and combination with other substances known to exert beneficial effect on chronic pain, like tricyclic antidepressants, opioids, dual norepinephrine/serotonin reuptake inhibitors and Ca^2+^ α_2_δ inhibitors like gabapentin or pregabalin. Thus, clinical trials investigating the effect of bitopertin or other GlyT1 inhibitors in human patients suffering from different forms of chronic pain are urgently required. These studies will reveal if GlyT1 inhibitors like bitopertin are a novel approach to treat chronic pain conditions.

## Ethics Statement

All animal experimental work presented in this study were approved by the animal health and care committees (regional council Ansbach, regional council Würzburg and regional council Düsseldorf).

## Author Contributions

AA contributed to planning of the study, validated and performed the majority of measurements, evaluated data and contributed to writing of the manuscript. EN contributed to planning of the study, validated and performed measurements and evaluated data. VK validated and performed measurements and evaluated data. HH contributed to planning of the study, evaluated data and contributed to writing of the manuscript. RW and VE initiated, planned and supervised the study, supervised laboratory measurements, evaluated the data and wrote the manuscript (together with other co-authors).

## Conflict of Interest Statement

A patent application was filed on the basis of the results described here by the Universities of Düsseldorf and Erlangen. Here, VE, HH, and RW were mentioned as inventors. Parts of the present work were performed by AA and VK in partial fulfillment of the requirement for obtaining the degree Dr. rer. nat. and Dr. med., respectively. The other author declares that the research was conducted in the absence of any commercial or financial relationships that could be construed as a potential conflict of interest.
